# Missense Mutations in the Unfoldase ClpC1 of the Caseinolytic Protease Complex Are Associated with Pyrazinamide Resistance in Mycobacterium tuberculosis

**DOI:** 10.1128/AAC.02342-16

**Published:** 2017-01-24

**Authors:** Michelle Yee, Pooja Gopal, Thomas Dick

**Affiliations:** Antibacterial Drug Discovery Laboratory, Department of Microbiology and Immunology, Yong Loo Lin School of Medicine, National University of Singapore, Singapore, Republic of Singapore

**Keywords:** ClpC1, Mycobacterium tuberculosis, caseinolytic protease, pyrazinamide, resistance

## Abstract

Previously, we showed that mutations in Mycobacterium tuberculosis *panD*, involved in coenzyme A biosynthesis, cause resistance against pyrazinoic acid, the bioactive component of the prodrug pyrazinamide. To identify additional resistance mechanisms, we isolated mutants resistant against pyrazinoic acid and subjected *panD* wild-type strains to whole-genome sequencing. Eight of the nine resistant strains harbored missense mutations in the unfoldase ClpC1 associated with the caseinolytic protease complex.

## TEXT

Pyrazinamide (PZA) is a critical component of the current first-line drug regimen to treat tuberculosis (TB). Inclusion of PZA in the regimen in the 1980s shortened the duration of therapy from 12 to 6 months (1). However, a 6-month regimen is still too lengthy for ensuring compliance, not only affecting cure rates, but also facilitating the development of drug resistance. Thus, shortening the treatment to 2 months or less is a major goal in TB drug development (2). Most new drug combinations under development include PZA, although its target(s) remains ill defined (3). Due to the clinically proven sterilizing activity of PZA, identifying its mechanism of action may provide clues to develop novel approaches for discovering shorter chemotherapeutic regimens.

PZA is a prodrug that must be converted to its bioactive form, pyrazinoic acid (POA). Prodrug conversion is carried out by the host's metabolism (4) and the bacterial amidase PncA, the inactivation of which causes PZA resistance *in vitro* (5). POA appears to have multiple bacterial targets. POA was proposed to act as an ionophore, causing intracellular acidification (6, 7), though this model was questioned (8). Biochemical and protein binding studies have identified at least two possible targets for POA, namely, fatty acid synthetase I (FASI) (9) and 30S ribosomal S1 protein (RpsA) (10). This suggests that POA may interfere with fatty acid synthesis and with *trans*-translation, which is a rescue mechanism that frees ribosomes stalled in translation. Recently, we demonstrated that at least two independent mechanisms of resistance to POA/PZA exist in Mycobacterium bovis BCG. First, high-level POA resistance is caused by missense mutations in aspartate decarboxylase *panD* (also reported in references 11 and 12), indicating that POA interferes with pantothenate and coenzyme A biosynthesis (13). Second, low-level POA resistance is caused by the loss of phthiocerol dimycocerosate (PDIM) virulence factor biosynthesis via frameshift mutations in the polyketide synthase genes *mas* and *ppsA* through *ppsE* (*ppsA-E*) (14). We also showed that the two resistance mechanisms were recapitulated in virulent Mycobacterium tuberculosis by whole-genome sequencing of 10 *in vitro*-isolated POA-resistant strains (14).

**FIG 1:**
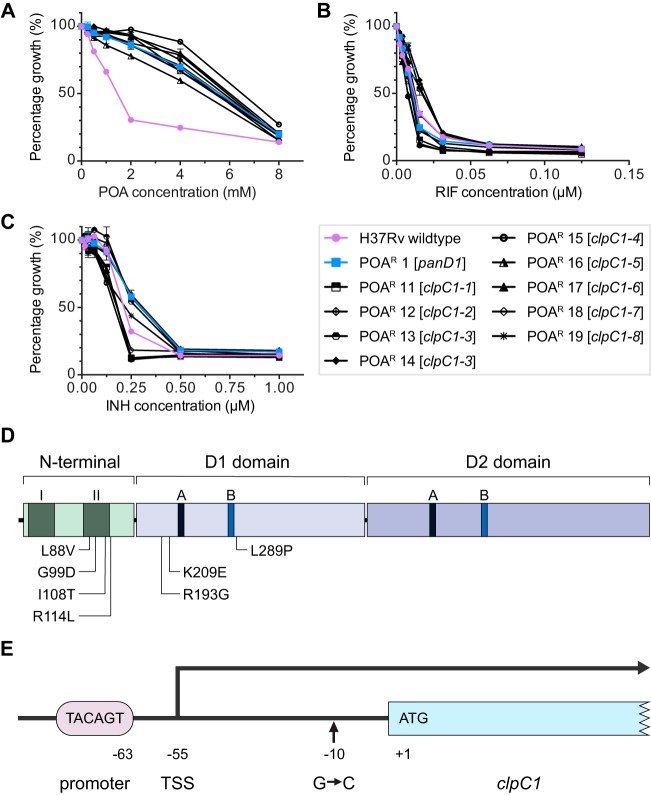
Characterization of pyrazinoic acid (POA)-resistant *panD* wild-type M. tuberculosis strains. Growth inhibition dose-response curves of 9 POA-resistant *panD* wild-type strains, POA^r^ 11 to 19, POA-sensitive wild-type M. tuberculosis H37Rv, and a representative POA-resistant *panD* mutant strain, POA^r^ 1, isolated previously (14), for (A) POA, (B) rifampin (RIF), and (C) isoniazid (INH). Experiments were carried out 3 times independently with technical replicates. Mean values and standard deviations from results of representative experiments are shown. (D) Location of 7 ClpC1 amino acid sequence polymorphisms in POA-resistant *panD* wild-type M. tuberculosis strains POA^r^ 12 to 19. ClpC1 domain organization is shown as described in reference 23. Within the N-terminal domain, two repeats are labeled I and II. A and B in the D1 and D2 domains indicate Walker A and Walker B motifs, respectively. (E) Location of the nucleotide sequence polymorphism G to C (−10) in the untranslated leader mRNA of *clpC1* in POA-resistant *panD* wild-type M. tuberculosis strain POA^r^ 11. The organization of the *clpC1* upstream region is shown as described in reference 20. A conserved TANNNT promoter motif (TACAGT) and the transcriptional start site (TSS), located 55 bp upstream of the *clpC1* coding sequence, are indicated (20). Refer to Table 1 for genotypes and phenotypes of strains.

Here, we asked whether additional “*panD*-like” mechanisms, i.e., high-level POA/PZA resistance mechanisms independent of *panD* mutations, can be identified in M. tuberculosis. To avoid selecting strains with loss-of-function mutations in the prodrug-activating amidase PncA, we selected M. tuberculosis H37Rv directly on 7H10 agar containing POA, i.e., on agar containing the bioactive form of PZA instead of the prodrug. We carried out spontaneous mutant selection, colony purification on respective POA-containing agar to verify drug resistance, and cryopreservation of resistant strains for four independent batches of M. tuberculosis cultures by plating on 2 mM or 4 mM POA as described previously (14). We chose these high concentrations of POA to avoid selecting low-level resistance mutations in *mas* and in *ppsA-E* (which can be selected on 1 mM POA [14]). We observed spontaneous mutation frequencies of 10^−4^ (2 mM POA) and 10^−5^ (4 mM POA), consistent with the frequencies reported previously by us (14) and by Lanoix et al. (15). The frozen stocks were expanded in 7H9 broth and genomic DNA was extracted (16). To identify *panD*-independent POA resistance mechanisms, we picked a total of 21 POA-resistant strains from the 4 independent selection experiments and showed by targeted sequencing that 12 of the strains carried expected (11, 12, 14) *panD* resistance mutations, while the remaining 9 strains harbored wild-type *panD* genes. Targeted *panD* sequencing was carried out by PCR amplification of the *panD* locus as described in reference 11 using Phusion polymerase (Thermo Scientific) followed by capillary sequencing of the PCR product, performed by AIT Biotech, Singapore, using BigDye Terminator chemistry. The 12 *panD* mutation-containing POA-resistant strains were excluded from this study. We determined the MICs to POA of the 9 POA-resistant *panD* wild-type M. tuberculosis strains to confirm resistance in liquid culture, and they were found to display 4-fold increases in MIC_50_ values, indicating high levels of POA resistance similar to that of the representative *panD* mutant strain POA^r^ 1 described previously in reference 14 (Table 1 and Fig. 1A). To verify that these strains displayed POA resistance specifically and not general antibiotic resistance, we measured MICs for rifampin and isoniazid and found that the strains showed wild-type-like susceptibility to these first-line TB drugs (Fig. 1B and C). We determined the MICs shown in Fig. 1A to C using the broth dilution method as described previously with minor modifications (4). The strains were grown to mid-log phase, spun down, and resuspended in fresh 7H9 medium adjusted to an optical density at 600 nm (OD_600_) of 0.1. Next, 100 μl of the cell suspension was added to wells containing 100 μl 2-fold serially diluted drugs in transparent flat-bottomed 96-well plates (Corning Costar) and sealed with Breathe-Easy membranes (Sigma-Aldrich). The plates were incubated for 7 days at 37°C with shaking at 80 rpm, and OD_600_ was measured using a spectrophotometer (Tecan Infinite M200 Pro). In addition to broth MICs, POA agar MICs were determined. The agar MIC was defined as the concentration of drug that suppressed colony formation when plating 10^4^ CFU from mid-log cultures on 7H10 agar plates (in 3 independent experiments) and incubating for 3 weeks at 37°C as described previously (17). The 9 POA-resistant *panD* wild-type strains displayed at least 4-fold increases in agar MIC for POA compared with that of wild-type M. tuberculosis H37Rv (Table 1). Furthermore, we demonstrated that each of the 9 strains was also resistant to the prodrug PZA using the Bactec MGIT 960 PZA susceptibility test (18) and by determining PZA agar MICs (19) as shown in Table 1. Altogether, the broth and agar MICs of the POA-resistant *panD* wild-type strains for POA and PZA revealed that all 9 strains showed (i) resistance to the bioactive form of PZA (POA) and the prodrug PZA itself, (ii) similar resistance levels, and (iii) resistance levels similar to the resistance level of the previously identified *panD* mutant strain (14). In other words, the resistance levels were “high” compared with the low level of resistance caused by mutations in the polyketide synthases Mas and PpsA-E (14).

**TABLE 1:** Sequence polymorphisms and POA and PZA broth and agar MICs of POA-resistant M. tuberculosis strains

M. tuberculosis H37Rv strain^*a*^	Mutations		POA^*b*^		PZA^*b*^	
	*clpC1*^*c*^	Other genes	MIC_50_ (mM) in broth	MIC^*d*^ (mM) in agar	S/R^*e*^	MIC^*d*^ (mM) in agar
Wild-type	—^*f*^	—	1.5	1	S	2
POA^r^ 1 (*panD1*)^*g*^ I	—	*panD*: Δ380A	6.0	>4	R	>4
POA^r^ 11 (*clpC1*-*1*) 1, I	G-10C^*h*^	—	5.5	>4	R	>4
POA^r^ 12 (*clpC1*-*2*) 2, II	C262G/Leu88Val	*mmpL7*: T534G/Asp178Glu	5.5	>4	R	>4
POA^r^ 13 (*clpC1*-*3*) 3, I	G296A/Gly99Asp	—	6.0	>4	R	>4
POA^r^ 14 (*clpC1*-*3*) 4, I	G296A/Gly99Asp	—	6.0	>4	R	>4
POA^r^ 15 (*clpC1*-*4*) 4, II	T323C/Ile108Thr	—	6.5	>4	R	>4
POA^r^ 16 (*clpC1*-*5*) 3, I	G341T/Arg114Leu	—	5.0	>4	R	>4
POA^r^ 17 (*clpC1*-*6*) 1, II	C577G/Arg193Gly	Rv3626c: G710T/Arg237Leu	6.0	>4	R	>4
POA^r^ 18 (*clpC1*-*7*) 2, II	A625G/Lys209Glu	—	6.0	>4	R	>4
POA^r^ 19 (*clpC1*-*8*) 3, I	T866C/Leu289Pro	*ppe47*: Ins14G; *yrbE4B*: G715A/Gly239Arg	6.0	>4	R	>4

a Mutants were isolated from four independent batches of bacterial cultures: 1 and 2, selected on Middlebrook 7H10 agar containing 0.5% glycerol; 3 and 4, selected on Middlebrook 7H10 agar without glycerol; I, mutants were selected on agar containing 2 mM POA; II, mutants were selected with 4 mM POA.

b Drug susceptibility tests were carried out 3 times independently and mean values are shown.

c Polymorphisms were identified by whole-genome sequencing and verified by targeted sequencing as described in the text.

d Maximum concentration of drug tested was 4 mM.

e BACTEC MGIT 960 test for susceptibility (S) or resistance (R) to 100 μg/ml PZA.

f —, not applicable.

g Isolated and described in reference 14.

h The polymorphism is 10 bp upstream of the *clpC1* start codon in the transcribed but untranslated leader sequence (see Fig. 1E).

To identify the genomic polymorphisms associated with resistance, the 9 POA/PZA-resistant *panD* wild-type strains were subjected to whole-genome sequencing. Whole-genome sequencing was performed on Illumina MiSeq as described previously (14). As was expected from selecting for resistance on agar containing high POA concentrations, we did not detect low POA resistance conferring *mas* or *ppsA-E* mutations (14) in the 9 strains. Table 1 shows that 8 of the 9 strains (POA^r^ 12 to 19) carried nonsynonymous single nucleotide polymorphisms in the coding sequences of ClpC1 (Rv3596c). These 8 *clpC1* missense mutation-harboring strains presented 7 different amino acid substitutions in the N-terminal and D1 domains of this 848-amino-acid protein (Fig. 1D), with one pair carrying identical amino acid changes (Table 1, POA^r^ 13 and 14). As the members of this pair were isolated from different selection experiments (i.e., from independently grown cultures), they likely represent independent mutational events and are not clonal in nature. The remaining POA-resistant strain, POA^r^ 11, showed a nucleotide polymorphism 10 bp upstream of the ClpC1 encoding sequence in the leader mRNA of the transcript (Table 1 and Fig. 1E) (20). Whether this mutation affects the expression level of the ClpC1 protein remains to be determined. The mutations in the *clpC1* gene were confirmed by targeted PCR sequencing using the following primer pairs: 5′-CGGCGACCTGACATTTGGCTACC-3′ and 5′-ACGCCTTCCCCTTCATGGATCAGG-3′ for strain POA^r^ 11 carrying a mutation upstream of ClpC1 encoding sequence, and 5′-ACATATGTTCGAACGATTTACCGACCGTGC-3′ and 5′-TGAATTCACCCATGTCAATCTGAATAAGCGC-3′ for the remaining strains with mutations in the ClpC1-encoding region. Taken together, all 9 POA/PZA-resistant *panD* wild-type M. tuberculosis strains harbored nucleotide polymorphisms in the *clpC1* locus. This result suggests that the observed mutations in this gene cause POA/PZA resistance.

Caseinolytic protein C (ClpC) can be found in both prokaryotes and eukaryotes. ClpC belongs to class I of the AAA+ (ATPases associated with a variety of cellular activities) superfamily containing one N-terminal and two nucleotide-binding domains (D1 and D2), the latter harboring the Walker A and Walker B motifs required for ATP binding and hydrolysis (21) (Fig. 1D). Bacterial ClpC proteins have been reported to function as molecular chaperones and specificity factors involved in determining substrates to be degraded by the caseinolytic protease complex (22). Similarly, in M. tuberculosis, the ClpC homolog ClpC1 self-associates to form oligomers displaying ATPase and molecular chaperone activities *in vitro* (23). ClpC1 works as an unfoldase in concert with the proteases ClpP1 and ClpP2 of the caseinolytic protease complex (24), and it was demonstrated that this degradative protease is essential for the viability of M. tuberculosis (25). Due to the critical role of this protease in survival and virulence, different components of this complex have been proposed as attractive therapeutic targets (26). Our POA-resistant strains harbor missense mutations in 2 different regions of the ClpC1 protein. We observed 4 different missense mutations in the N-terminal domain of ClpC1, with 3 located in the N-terminal repeat II (as annotated by reference 23) and the fourth mutation just outside this repeat (Fig. 1D). While the exact role of the N-terminal domain of M. tuberculosis ClpC1 is unclear, it is interesting to note that it acts as the binding site of several novel antimycobacterials, including cyclomarin (27), lassomycin (28), and ecumicin (29). In other prokaryotes, the N-terminal domain of ClpC is the site for interacting with adaptor proteins, either acting as the binding site or aiding in substrate recognition (21, 30). The other 3 missense mutations are located in the D1 domain, flanking the Walker A and Walker B motifs (Fig. 1D).

It remains to be determined whether the observed missense mutations in the coding regions of *clpC1* cause POA/PZA resistance via a direct mechanism, for instance, by preventing binding of the drug to the ClpC1 protein, or an indirect mechanism, for instance, by affecting the substrate selectivity of the ClpC1 unfoldase and therefore the level of some proteins targeted for degradation by the caseinolytic protease complex.

Similar to the POA/PZA-associated resistance mutations in *panD* isolated *in vitro* (11, 12, 14), *clpC1* polymorphisms appear to not be strongly associated with PZA resistance in clinical isolates of M. tuberculosis. In the Genome-wide Mycobacterium tuberculosis variation (GMTV) database (31), we did not find any strains with the *clpC1* polymorphisms observed in our POA-resistant strains. It has been shown that *clpC1* is essential for growth *in vitro* (32, 33) and for survival within macrophages (34). Whether the absence of our POA/PZA resistance-causing mutations in clinical isolates results from a loss of *in vivo* fitness is under investigation in mouse infection studies.

In conclusion, we add here to the growing list of POA/PZA candidate targets and resistance mechanisms, including fatty acid synthetase I (FASI), ribosomal protein S1 (RpsA), the aspartate decarboxylase PanD, and the polyketide synthases Mas and PpsA-E, by demonstrating that missense mutations in the unfoldase/ATPase ClpC1 of the caseinolytic protease complex are associated with POA and PZA resistance. This provides further support for a working model suggesting that the excellent sterilizing activity of PZA may be due, in part, to it being a “dirty drug”, i.e., this small “fragment-like” antimycobacterial can hit multiple targets and pathways inside the tubercle bacillus (35).
